# SLC7A11 Reduces Laser-Induced Choroidal Neovascularization by Inhibiting RPE Ferroptosis and VEGF Production

**DOI:** 10.3389/fcell.2021.639851

**Published:** 2021-02-18

**Authors:** Xiaohuan Zhao, Min Gao, Jian Liang, Yuhong Chen, Yimin Wang, Yuwei Wang, Yushu Xiao, Zhenzhen Zhao, Xiaoling Wan, Mei Jiang, Xueting Luo, Feng Wang, Xiaodong Sun

**Affiliations:** ^1^Department of Ophthalmology, Shanghai General Hospital, Shanghai Jiao Tong University School of Medicine, Shanghai, China; ^2^National Clinical Research Center for Eye Diseases, Shanghai, China; ^3^Shanghai Key Laboratory of Fundus Diseases, Shanghai, China; ^4^Shanghai Engineering Center for Visual Science and Photomedicine, Shanghai, China; ^5^The Center for Microbiota and Immunological Diseases, Shanghai General Hospital, Shanghai Institute of Immunology, Department of Immunology and Microbiology, Shanghai Jiao Tong University School of Medicine, Shanghai, China

**Keywords:** SLC7A11, RPE, ferroptosis, VEGF, CNV

## Abstract

In age-related macular degeneration (AMD), one of the principal sources of vascular endothelial growth factor (VEGF) is retinal pigment epithelium (RPE) cells under hypoxia or oxidative stress. Solute carrier family 7 member 11 (SLC7A11), a key component of cystine/glutamate transporter, regulates the level of cellular lipid peroxidation, and restrains ferroptosis. In our study, we assessed the role of SLC7A11 in laser-induced choroidal neovascularization (CNV) and explored the underlying mechanism. We established a mouse model of CNV to detect the expression level of SLC7A11 and VEGF during disease progression. We found the expression of the SLC7A11 protein in RPE cells peaked at 3 days after laser treatment, which was correlated with the expression of VEGF. Intraperitoneal injection of SLC7A11 inhibitor expanded the area of CNV. We examined functional proteins related to oxidative stress and Fe^2+^ and found laser-induced ferroptosis accompanied by increased Fe^2+^ content and GPX4 expression in the RPE-choroidal complex after laser treatment. We verified the expression of SLC7A11 in the ARPE19 cell line and the effects of its inhibitors on cell viability and lipid peroxidation *in vitro*. Application of SLC7A11 inhibitor and SLC7A11 knockdown increased the level of lipid peroxidation and reduced the cell viability of ARPE19 which can be rescued by ferroptosis inhibitors ferrostatin-1 (Fer-1) and liproxstatin-1 (Lip-1). Conversely, SLC7A11 overexpression induced resistance to erastin or RSL3-induced ferroptosis. Moreover, we tested the possible regulatory transcription factor NF-E2-related factor 2 (NRF2) of SLC7A11 by Western blot. Knock-down of NRF2 decreased the expression of SLC7A11. Our study suggests that SLC7A11 plays a key role in the laser-induced CNV model by protecting RPE cells from ferroptosis. SLC7A11 provides a new therapeutic target for neovascular AMD patients.

## Background

Age-related macular degeneration (AMD) is the leading cause of irreversible vision loss among the elderly in developed countries. It is estimated that there will be 288 million AMD patients by 2040 around the world (Wong et al., 2014). AMD has two clinical subtypes: geographic atrophy (dry) types and neovascular (wet) types. Neovascular AMD (nAMD) causes severe and rapid vision loss in 80% of patients who have it. This loss is characterized by abnormal choroidal neovascularization (CNV) (Ishikawa et al., 2016). CNV is the growth of new blood vessels that originate from the choroid through a break in Bruch’s membrane. The blood vessels invade the subretinal pigment epithelial and subretinal space, which causes exudation, hemorrhage, and secondary fibrovascular scarring (Al Gwairi et al., 2016; Al-Zamil and Yassin, 2017).

Multiple regulatory factors mediate the formation and development of CNV, especially the development of new blood vessels (angiogenesis) induced by vascular endothelial growth factor (VEGF) (Nowak et al., 2003). It is believed that VEGF and its receptor are the most important regulators of CNV (Shibuya, 2011). So, targeted VEGF therapy is a primary treatment for nAMD. However, some patients with AMD do not respond to targeted therapy. Because of the short half-life of anti-VEGF drugs, many AMD patients need long-term or even life-long treatment. This has adverse effects such as retinal atrophy (Xiao et al., 2019), and the patients’ safety and the economic burden on them must be considered. Besides directly blocking VEGF or inhibiting VEGF signals, it is also worth determining if regulating upstream signals indirectly inhibits the production of VEGF (Al-Latayfeh et al., 2012).

A principal source of VEGF is retinal pigment epithelium (RPE) cells under hypoxia and oxidative stress (Kannan et al., 2006; Paeng et al., 2015; Arjamaa et al., 2017). RPE cells play an important role in phagocytizing the outer segment of the photoreceptor, which is highly enriched with polyunsaturated fatty acids and leaves RPE particularly susceptible to lipid peroxidation (Yang et al., 2006). Moreover, some studies have shown that the development of AMD involves lipid peroxidation and iron metabolism (Yang and Stockwell, 2016; Sun et al., 2018). It is unclear how lipid reactive oxygen species (ROS) and iron dysregulation contribute to CNV.

Ferroptosis is a non-apoptotic, iron-dependent regulated cell death, driven by lipid peroxide accumulation (Yang and Stockwell, 2016). Ferroptosis results in morphological changes, including mitochondrial shrinkage and increased mitochondrial membrane density. Ferroptosis can be induced by glutathione peroxidase 4 (GPX4) inhibitors (Yu et al., 2017). SLC7A11, a key component of cystine/glutamate transporter on the membrane, promotes cystine uptake, and glutathione synthesis (Koppula et al., 2018). Pharmacological inhibition SLC7A11 causes cystine depletion and the accumulation of lipid peroxide.

In this study, we focused on ferroptosis and the regulation of iron metabolism in CNV. We designed experiments to study whether oxidative stress, regulated by SLC7A11, played a role in the progression of CNV. The results showed that oxidative-stress-related protein and SLC7A11 increased in a mouse model of laser-induced CNV. Next, we inhibited SLC7A11 in both CNV mouse and ARPE19 cell to further clarify its protective role and explore its related molecular mechanism. We found that SLC7A11 could serve as a new therapeutic target to AMD.

## Materials and Methods

### Cell Culture

The ARPE19 cell line were cultured in DMEM/F-12 medium added to 10% fetal bovine serum (Sigma-Aldrich, St. Louis, MO, United States) and 1% penicillin/streptomycin (GIBCO). They were cultured at 37°C with 5% CO_2_ and treated with different reagents according to experimental design: H_2_O_2_ (Sigma) and SAS (sulfasalazine, Sigma).

### Animals

Female C57BL/6 mice, aged 6–8 weeks (Shanghai, China) were used. The water and food consumed by all mice was sterilized. Each experimental group had five or six mice for laser-induced CNV. All animal experiments were approved by the Ethics Committee of Shanghai Jiao Tong University, Shanghai, China, and they were conducted in compliance with the Association for Research in Vision and Ophthalmology Statement for the Use of Animals in Ophthalmic and Vision Research.

### GSH Determination

ARPE19 intracellular GSH was detected using a GSH and GSSG Assay Kit (Beyotime, S0053) according to the manufacturer’s instructions. The absorbance was measured at 405 nm with a microplate reader (Infinite F50 microplate reader, Swiss). The glutathione and GSSG content of the sample were calculated according to the standard curve, and the GSH content was obtained by the formula: GSH = total glutathione-GSSG × 2 (μM). Experiments were done in three parallel wells and repeated at least twice.

### Lipid Peroxidation Detection

BODIPY^TM^ 581/591 C11 (Thermo Fisher, D3861) was used to measure ARPE19 cell lipid peroxidation as reported (Sun et al., 2018). BODIPY was incorporated into cells seeded in a six-well plate for 30 min at a final concentration of 2 μM. The cells were washed three times with PBS. Then flow cytometry (Beckman Coulter, United States) and fluorescence microscopy were used to detect the changes of the probe emission. When lipid peroxidation occurs, the emission of BODIPY changes from red to green (Christova et al., 2004). Experiments were done in three parallel wells and repeated at least twice.

### Measurement of Cell Viability

A CCK-8 kit (Beyotime) was used to detect cell viability. ARPE19 was seeded in a 96-well plate and incubated at 37°C. When the cell density was 80–90% of the well, the cells were washed with PBS and treated with CCK-8 reagent for 1 h at 37°C. Absorbance was measured at 450 nm using a microplate reader (Infinite F50 microplate reader, Swiss). Experiments were done in six parallel wells and repeated at least twice.

### Western Blot

Retinal pigment epithelium-choroidal complex or RPE cells were collected and measured with the Pierce bicinchoninic acid (BCA) assay. Approximately 10 μg protein were added to SDS-PAGE gels ranging from 8 to 15% and transferred onto a PVDF membrane (Merck Millipore, Billerica, MA, United States). The membranes were blocked with Tris-buffered saline Tween-20 (TBST) containing 5% skim milk for 1 h. Then they were incubated overnight at 4°C in primary antibody: xCT (Novus Biologicals, NB 300-318, 1:1000), NRF2 (Proteintech, 1:1000), HO-1 (Proteintech, 1:1000), GCLC (Proteintech, 1:1000), NQO1 (Proteintech, 1:1000), GPX4 (Abcam, 1:1000), GAPDH (Proteintech, 1:1000). The membranes were washed with TBST and incubated with secondary antibodies conjugated with horseradish peroxidase (HRP) (Proteintech, 1:5000) for 1 h. Then the plots were exposed to a molecular imaging system (Amersham Imager 600, GE Healthcare, Buckinghamshire, United Kingdom).

### Real-Time PCR Analysis

Total RNA was isolated according to the RNAsimple Total Kit protocol (Tiangen Biotech, Beijing, China) and quantified by NanoDrop 2000c spectrophotometer (Thermo Fisher Scientific, Wilmington, DE, United States). cDNA was synthesized according to the RT Master Mix protocol (Takara Bio Inc., Dalian, China). The primers were: mouse *Slc7a11*, 5′- TGGGTGGAACTGCTCGTAAT -3′ (forward) and 5′- AGGATGTAGCGTCCAAATGC -3′ (reverse); mouse *Nrf2*, 5′- CTTTAGTCAGCGACAGAAGGAC-3′ (forward) and 5′-AGGCATCTTGTTTGGGAATGTG-3′ (reverse); mouse *GAPDH*, 5′- TGACCTCAACTACATGGTCTACA -3′ (forward) and 5′- CTTCCCATTCTCGGCCTTG -3′ (reverse); human *SLC7A11*, 5′- GCGTGGGCATGTCTCTGAC -3′ (forward) and 5′-GCTGGTAATGGACCAAAGACTTC-3′ (reverse); human *NRF2*, 5′- TCTGACTCCGGCATTTCACT-3′ (forward) and 5′- GGCACTGTCTAGCTCTTCCA -3′ (reverse); human *VEGFA*, 5′-AGGGCAGAATCATCACGAAGT-3′ (forward) and 5′-AGGGTCTCGATTGGATGGCA-3′ (reverse); human *GAPDH*, 5′-TGTGGGCATCAATGGATTTGG-3′ (forward) and 5′-ACACCATGTATTCCGGGTCAAT-3′ (reverse).

### *NRF2* RNA Interference

ARPE19 was transfected with double-stranded siRNA to knock down *NRF2* by Lipofectamine 3000 (Thermo Fisher Scientific). The sequences were: si-*NRF2*-1 5′-UAAAGUGGCUGCUCAGAAUUU-3′, si-*NRF2*-2 5′-GACAGAAGUUGACAAUUAUTT-3′, si-*NRF2*-3 5′-CCAGAACACUCAGUGGAAUTT-3′. Double-stranded siRNAs were synthesized by Shanghai GenePharma (Shanghai, China).

### Laser-Induced CNV in Mice

C57BL/6J (6–8 weeks old) mice were anesthetized by intraperitoneally injecting 1.5% sodium pentobarbital (100 μl/20g). 0.5% tropicamide and 0.5% noradrenaline hydrochloride eye drops were dripped into the eyes of the mice to dilate the pupils. Laser photocoagulation (350 Mw, 50 ms) was performed in the 2, 5, 8, and 11 o’clock position around the optic disk and kept away from blood vessels. It was focused on RPE, and it disturbed Bruch’s membrane when bubbles were observed.

### Fundus Fluorescent Angiography (FFA)

0, 1, 3, 7, and 10 days after laser injury, FFA was performed before sacrifice. After being anesthetized, the pupils of mice were dilated and 2% fluorescein sodium (Fluorescite; Alcon, Tokyo, Japan) was injected intraperitoneally. FFA images were made at intervals of 2 min. The FFA images were analyzed using ImageJ software (NIH, United States).

### Immunofluorescence Staining

Immunofluorescence stain assay was performed on sections of the eyes (10 μm in thickness), made with a microtome. After fixation, the samples were blocked with PBS containing 0.3% Triton X-100 and 5% goat serum albumin (Beyotime) for 1 h. Then the sections were incubated overnight at 4°C with primary antibodies against SLC7A11 (CST, 1:1000), isolectin (Santa Cruz Biotechnology, 1:1000), RPE65 (Abcam, 1:1000). The sections were stained for 1 h with Alexa Fluor 594 or 488-conjugated secondary antibodies (Proteintech, 1:1000). This step was not required for the immunofluorescence of isolectin. The retinal sections were visualized using a confocal microscope (Leica, United States).

### *SLC7A11* shRNA Plasmids Construction

The pLKO.1-*SLC7A11* shRNA oligos were: shRNA-1 CCTGTCACTATTTGGAGCTTT, shRNA-2 CCTGCGTATTATCTCTTTATT, and shRNA-3 CCCTGGAGTTATGCAGCTAAT. The pLKO.1-*SLC7A11* and scrambled shRNA with packing plasmids pSPA and pMD2G (Addgene) were transfected into HEK293 packing cells using Lipofectamine 3000 (Thermo Fisher Scientific). *SLC7A11*, shRNA, or scrambled shRNA were introduced into ARPE19 cells by transfection with lentiviral supernatants. RT-PCR and Western blot were used to select stable transfected sequences with a certain knockdown efficiency for subsequent experiments.

### Cloning and Transfection

SLC7A11 plasmid was constructed by using the methods described (Nazari et al., 2014; Nasca et al., 2017). Briefly, the SLC7A11 cDNA was subcloned into pAAV-CMS vector. New recombinant plasmids were termed as AAV-SLC7A11. Then the AAV- SLC7A11 was transfected into ARPE19 cells with the Lipofectamine 3000 (Thermo Fisher Scientific). 6 h after transfection, the cells were cultured in fresh medium containing erastin (Sigma-Aldrich) or RSL3 (CAYMAN) for 24 h, then the culture medium was replaced by the fresh medium. The cell viability was measured by the CCK8 48 h after transfection.

### PI Staining

Staining with PI (81845, Sigma) was carried out according to the manufacturer’s instructions. Briefly, cells were suspended and stained with PI at a final concentration of 30 μM for 30 min (Rosenberg et al., 2019). The samples were counterstained with DAPI for fluorescence observation.

### Iron Assay

Fe^2+^ was found in the RPE-choroidal complex by using an iron assay kit (Sigma-Aldrich, MAK025) according to the manufacturer’s instructions. Absorbance was measured at 595 nm using a microplate reader (Infinite F50 microplate reader, Swiss).

### Statistical Analysis

Statistical analysis was performed using SPSS 21.0 software (GraphPad Prism, San Diego, CA, United States). Data were expressed as the mean ± SD from at least three biological replicates. The differences between the two groups were analyzed by a Student’s *t*-test. One-way ANOVA analysis was used to identify differences when there were three or more groups. A value of *p* < 0.05 was considered statistically significant.

## Results

### Laser-Induced CNV Vascular Leakage and Dynamic Changes of VEGFA

**FIGURE 1:**
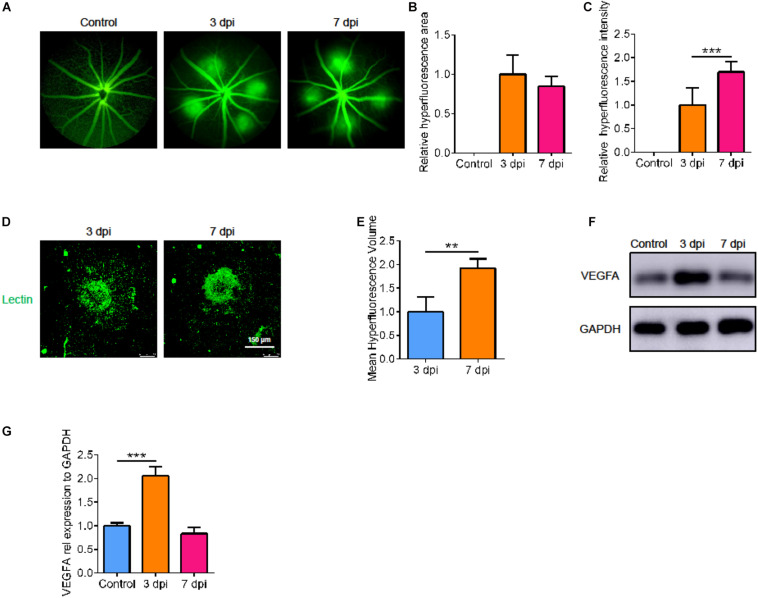
Laser-induced CNV vascular leakage and dynamic changes of VEGFA. **(A)** Fluorescence imaging of mice at various points in time after laser treatment. **(B,C)** The average area and intensity of fluorescence imaging after laser treatment by Image-J (*n* = 6). **(D,E)** Laser spot size at 3 dpi and 7 dpi by immunofluorescence and Image-J (*n* = 6). **(F,G)** The VEGFA protein changes dynamically at the corresponding point in time by Western blot (*n* = 3). Bar graphs show mean ± SD. *P*-values: T-test and One-way ANOVA analysis. (***p < 0.001, **p < 0.01). Scale bar = 150 μm.

To understand the natural process of CNV, we measured the leakage area and volume of CNV fluorescence at various points in time after laser treatment. Compared with 3 days after laser treatment, there was no significant difference in the fluorescence area at 7 days, but the fluorescence intensity increased (Figures 1A–C, *p* < 0.05). In addition, by immunofluorescence, we found that the volume of neovascularization at 7 days post injury (dpi) was larger than that at 3 dpi (Figures 1D,E, *p* < 0.05). So, we measured the size of the CNV at 7 dpi. We then detected the level of VEGFA at the corresponding point in time by Western blot. After laser treatment, VEGFA expression was upregulated; it peaked at 3 dpi, then decreased to normal at 7 dpi (Figures 1F,G, *p* < 0.05).

### The Expression of SLC7A11 in the Natural Course of Laser-Induced CNV in Mice

**FIGURE 2:**
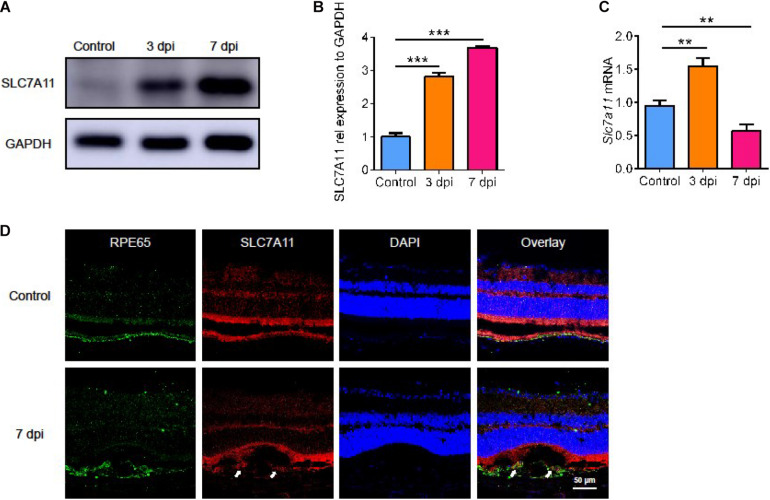
The expression of SLC7A11 in the natural course of laser-induced CNV in mice. **(A,B)** The level of SLC7A11 protein at various points in time after laser by Western blot (*n* = 3). **(C)** The level of *Slc7a11* mRNA after laser by RT-PCR (*n* = 6). **(D)** The location of SLC7A11 (white arrow) by immunofluorescence. Bar graphs show mean ± SD. *P*-values: one-way ANOVA analysis (***p < 0.001, **p < 0.01). Scale bar = 50 μm.

Next, we evaluated the level of SLC7A11 after laser treatment. Both SLC7A11 protein (Figures 2A,B, *p* < 0.05) and mRNA (Figure 2C, *p* < 0.05) rose after laser treatment. The mRNA level reached a peak at 3 dpi and fell back at 7 dpi. This showed that SLC7A11 can respond to laser-induced damage in the early stage. The SLC7A11 protein level gradually increased with time, and it peaked at 7 dpi. We used immunofluorescence to identify cells with high SLC7A11 expression. SLC7A11 was highly expressed and co-stained with RPE cells after 7 days of CNV; while at 0 dpi, almost no SLC7A11 signal was detected (Figure 2D). This is consistent with the protein level. In addition, we found that RPE cells became larger and more irregular after laser treatment. This suggests that with the progress of CNV, the RPE layer was destroyed, and the morphology and size changed. In response to injury, RPE cells produced SLC7A11 which increases with time.

### The Level of Oxidative Stress in Laser-Induced CNV

**FIGURE 3:**
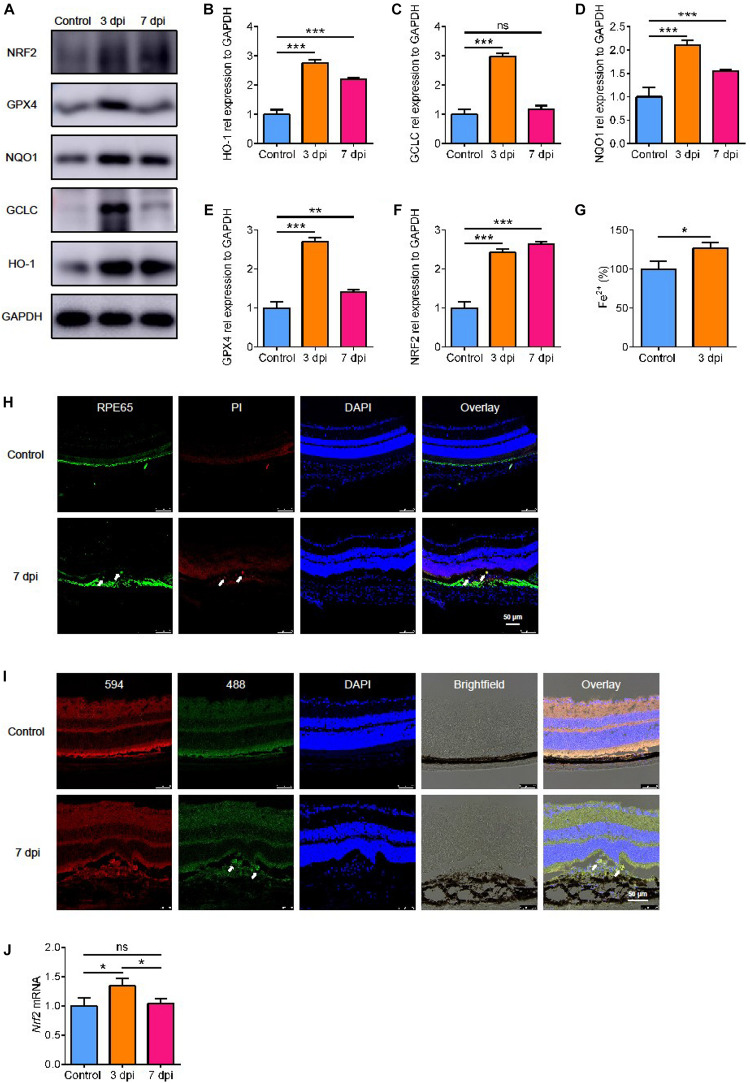
The expression of proteins involved in oxidative stress. **(A–F)** The expression of proteins involved in oxidative stress detected by Western blot (*n* = 3). **(G)** The content of Fe^2+^ in the RPE-choroidal complex after CNV (*n* = 6). **(H)** Cell death (white arrow) after laser by PI staining. **(I)** The level of lipid peroxidation (white arrow) after laser with C11 BODIPY 581/591. **(J)** The expression of *Nrf2* mRNA after laser by RT-PCR (*n* = 6). Bar graphs show mean ± SD. *P*-values: *t*-test and one-way ANOVA analysis (***p < 0.001, **p < 0.01, *p < 0.05). Scale bar = 50 μm.

SLC7A11 is a key component of glutamate/cystine transporter on the cell membrane. It promotes cystine uptake and glutathione biosynthesis, protecting cells from oxidative stress and ferroptotic death (Koppula et al., 2018). To verify the level of oxidative stress after laser treatment, we detected the expression of three proteins involved in antioxidant effects: heme oxygenase-1 (HO-1), glutamate-cysteine ligase catalytic subunit (GCLC), and NAD(P)H quinone dehydrogenase 1 (NQO1). All of them increased and reached a peak at 3 dpi and decreased slightly by 7 dpi (Figures 3A–D, *p* < 0.05). This suggests that there is oxidative stress in the process of laser-induced neovascularization.

### Ferroptosis Was Induced in RPE Cells of CNV *in vivo*

Because of the high level of oxidative stress, we looked for signs of damage related to oxidative stress. We found that glutathione peroxidase 4 (GPX4), an antioxidant enzyme, increased significantly by 3 days after CNV, and it decreased after 7 days, the same as HO-1 and NQO1 (Figures 3A,B,D,E, *p* < 0.05). GPX4 senses oxidative stress and converts lipid hydroperoxides into non-toxic lipid alcohols (Bersuker et al., 2019) to prevent ferroptosis (Seiler et al., 2008). To determine if ferroptosis occurred after CNV, we measured the iron content in the choroid. We found that the content of Fe^2+^ in the RPE-choroidal complex after CNV was higher in CNV group than in the control group (Figure 3G, *p* < 0.05). We used PI staining to measure cell death and BODIPY^TM^ 581/591 C11 to test lipid peroxidation. After CNV treatment, RPE cells death occurred with high level of lipid peroxidation (Figures 3H,I). Therefore, we speculated that choroidal ferroptosis occurs after CNV, and the compensatory increase of SLC7A11 and GPX4 in RPE is to reduce the ferroptosis of cells. In addition, a possible regulatory gene upstream of SLC7A11, *Nrf2*, was also upregulated at both protein and mRNA levels after laser treatment, and this lasted at least to 7 dpi (Figures 3A,F,J, *p* < 0.05).

### The Cell Viability and Lipid Peroxidation Level of RPE Cells After SLC7A11 Inhibition

**FIGURE 4:**
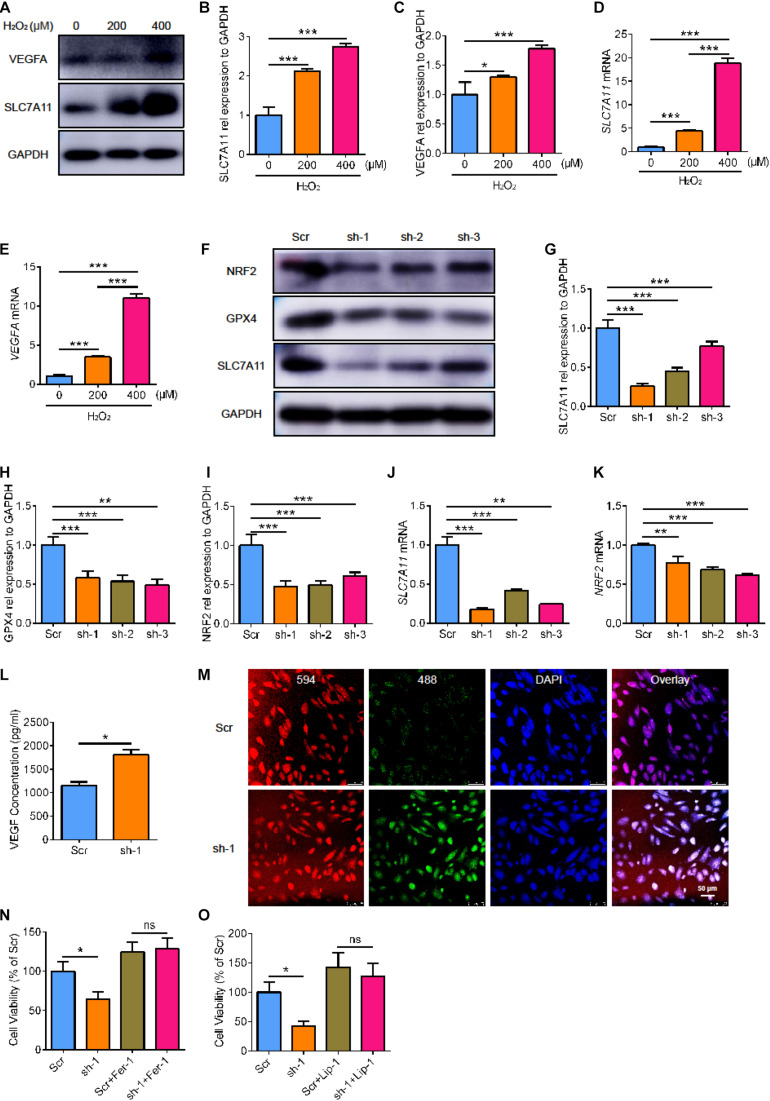
The cell viability and lipid peroxidation level of *SLC7A11*^*KD*^ ARPE19. **(A–C)** The protein expression of SLC7A11 and VEGFA in ARPE19 with different concentration of H_2_O_2_, including 0, 200, 400 μM (*n* = 3). **(D,E)** The mRNA level of *SLC7A11* and *VEGFA* in ARPE19 with different concentration of H_2_O_2_ (*n* = 6). **(F–I)** The expression of SLC7A11, GPX4, and NRF2 protein using shRNA to knock down *SLC7A11* (*n* = 3). **(J,K)** The mRNA of *SLC7A11* and *NRF2* using shRNA to knock down *SLC7A11* (*n* = 6). **(L)** The concentration of VEGF of *SLC7A11*^*KD*^ ARPE19 by ELISA (*n* = 5). **(M)** The level of lipid peroxidation of *SLC7A11*^*KD*^ ARPE19 by BODIPY^TM^ 581/591 (*n* = 5). **(N,O)** The cell viability of *SLC7A11*^*KD*^ ARPE19 with the treatment of Fer-1 (1000 nM) and Lip-1 (20 μM) (*n* = 6). Bar graphs show mean ± SD. *P*-values: T-test and one-way ANOVA analysis. (***p < 0.001, **p < 0.01, *p < 0.05). Scale bar = 50 μm.

To further clarify the role of SLC7A11 in RPE, we used ARPE19 cells treated with H_2_O_2_ for 24 h. We found that the expression of VEGFA and SLC7A11 increased as the concentration of H_2_O_2_ increased (Figures 4A–E, *p* < 0.05). To investigate the critical effect of SLC7A11, we used short hairpin RNA (shRNA) to knock down the expression of *SLC7A11* in ARPE19. The *SLC7A11*^*KD*^ ARPE19 showed a low protein and mRNA expression of SLC7A11 (Figures 4F,G,J, *p* < 0.05). Interestingly, we found that knocking down *SLC7A11* led to lower expression of NRF2 at both protein and mRNA levels compared to control group (Figures 4F,I,K, *p* < 0.05). This suggests that SLC7A11 regulates NRF2 expression at the transcription and translation levels. After exposure to H_2_O_2_, *SLC7A11*^*KD*^ ARPE19 expressed less GPX4 (Figures 4F,H, *p* < 0.05). This indicated that SLC7A11 regulated the expression of GPX4. In *SLC7A11*^*KD*^ ARPE19 supernatant, we observed upregulation of VEGF which indicated *SLC7A11* loss has an effect on ARPE19 expression of VEGF (Figure 4L, *p* < 0.05). We next detected oxidation by the ratiometric fluorescent lipid peroxidation sensor BODIPY^TM^ 581/591. *SLC7A11*^*KD*^ ARPE19 exhibited higher levels of lipid peroxidation compared to normal cells, which suggests that SLC7A11 inhibited lipid peroxidation (Figure 4M and Supplementary Figure 1A). We observed lower cell viability in *SLC7A11*^*KD*^ ARPE19 cells rather than control cells (Figure 4N,O, *p* < 0.05). To test whether ARPE19 cells undergo ferroptotic death upon *SLC7A11* inhibition, we treated ARPE19 with the potent ferroptosis inhibitors ferrostatin-1 (Fer-1) and liproxstatin-1 (Lip-1) (Zilka et al., 2017). We found that ferroptosis-rescuing antioxidant Fer-1 and Lip-1 rescued the decreased cell viability caused by *SLC7A11* deletion using CCK-8 and PI staining (Figures 4N,O and Supplementary Figure 1B, *p* < 0.05). This shows that *SLC7A11*^*KD*^ ARPE19 undergo ferroptotic death.

**FIGURE 5:**
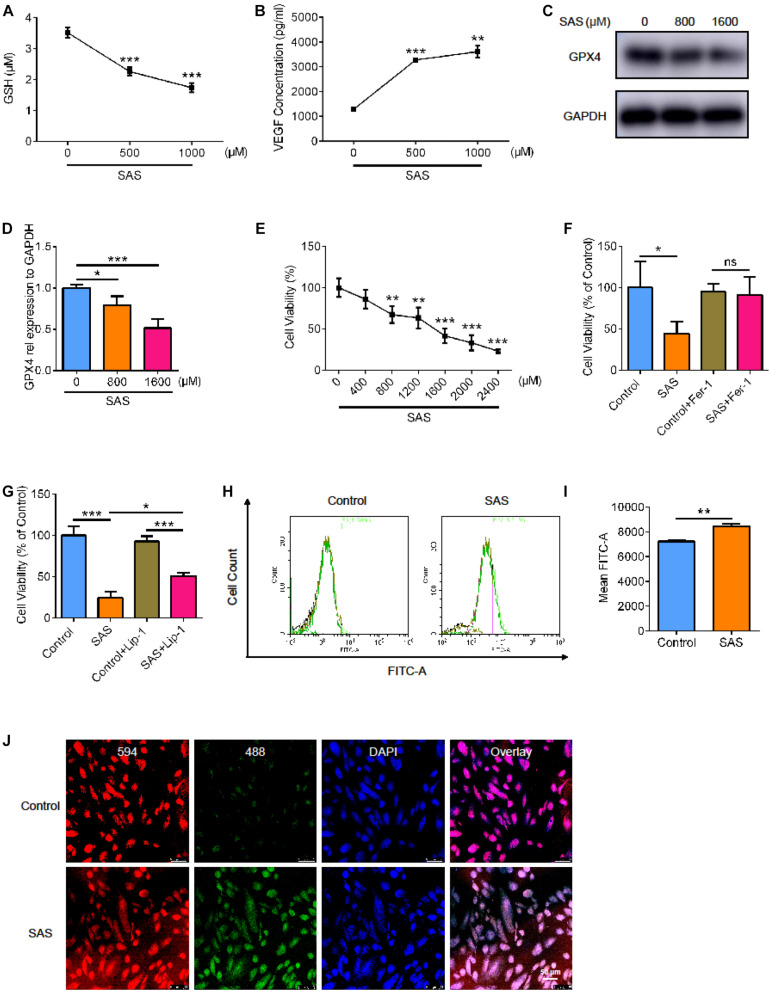
The effect of SLC7A11 inhibitor on ARPE cell line. **(A)** The GSH of ARPE19 with different concentration of SAS (*n* = 5). **(B)** The concentration of VEGF of ARPE19 with SAS stimulation by ELISA (*n* = 5). **(C,D)** The protein expression of GPX4 with different concentration of SAS (*n* = 3). **(E)** The cell viability of ARPE19 with different concentration of SAS (*n* = 5). **(F,G)** The cell viability of ARPE19 with treatment of Fer-1 (1000 nM) or Lip-1 (20 μM) after stimulation of 1600 μM SAS (*n* = 6). **(H–J)** The level of lipid peroxidation under SAS stimulation by BODIPY^TM^ 581/591 (*n* = 5). Bar graphs show mean ± SD. *P*-values: one-way ANOVA analysis and *t*-test (***p < 0.001, **p < 0.01, *p < 0.05). Scale bar = 50 μm.

Next, we used the SLC7A11 inhibitor, sulfasalazine (SAS), to stimulate the ARPE19 cell line. We examined the level of glutathione (GSH) and found that as the concentration of SAS increased, the level of GSH decreased. This indicated that SAS inhibited the function of SLC7A11 (Figure 5A, *p* < 0.05). We detected VEGF in cell supernatant by enzyme linked immunosorbent assay (ELISA) and found that the treatment of SAS increased the concentration of VEGF (Figure 5B, *p* < 0.05). This suggests that inhibition of SLC7A11 promoted the expression of VEGF. As the concentration of SAS increased, the expression of GPX4 in ARPE decreased, indicating a decrease in antioxidant capacity (Figures 5C,D, *p* < 0.05). We noticed that cell viability decreased as SAS concentration increased, which could be rescued by Fer-1 (Figures 5E,F and Supplementary Figure 2A, *p* < 0.05). However, Lip-1 only partially alleviates the decline in cell viability caused by SAS, because of the possible toxic effect of SAS itself, which has been reported in previous studies (Figure 5G and Supplementary Figure 2A, *p* < 0.05) (Linares et al., 2011; Li et al., 2020; Zheng et al., 2020). Similarly, SAS increased the ARPE19 lipid peroxidation level, detected by flow and immunofluorescence (Figures 5H–J and Supplementary Figure 2B, *p* < 0.05). This shows that inhibition of SLC7A11 enhanced lipid peroxidation and promoted ARPE19 to undergo ferroptosis.

### SLC7A11 Overexpression Induces Ferroptosis Resistance

**FIGURE 6:**
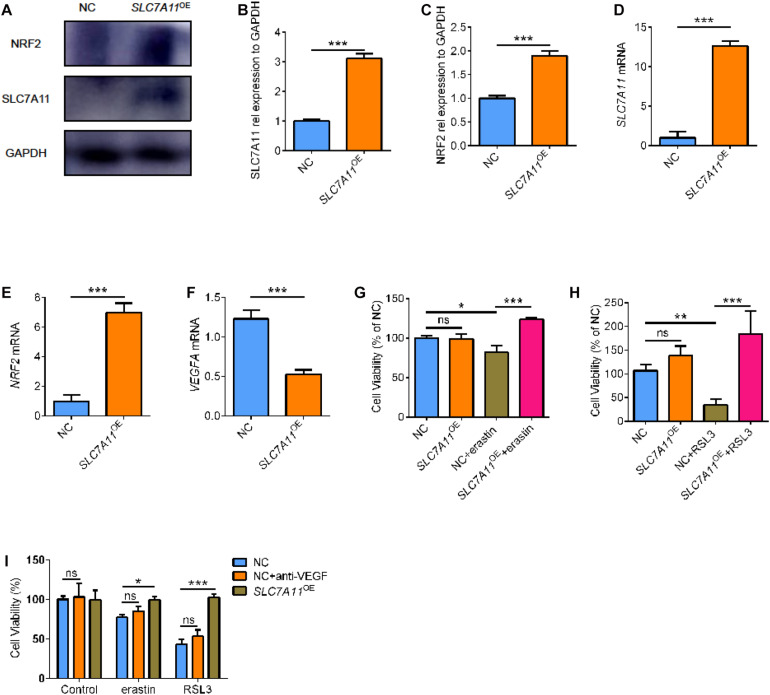
The cell viability of *SLC7A11*^*OE*^ ARPE19. **(A–C)** SLC7A11 and NRF2 protein in *SLC7A11*^*OE*^ ARPE19 were evaluated by Western blot (*n* = 3). **(D–F)** *SLC7A11*, *NRF2*, and *VEGFA* mRNA in *SLC7A11*^*OE*^ ARPE19 were evaluated by RT-PCR (*n* = 6). **(G,H)** The cell viability of *SLC7A11*^*OE*^ ARPE19 with the stimulation of erastin (200 μM) or RSL3 (20 μM) (*n* = 5). **(I)** The cell viability of anti-VEGF treatment and *SLC7A11*^*OE*^ ARPE19 with the stimulation of erastin (200 μM) or RSL3 (20 μM) (*n* = 5). Bar graphs show mean ± SD. *P*-values: one-way ANOVA analysis and *t*-test (***p < 0.001, **p < 0.01, *p < 0.05).

To further investigate the functional relevance of SLC7A11 in RPE biology, SLC7A11 plasmid was transfected into ARPE19 cells (*SLC7A11*^*OE*^). SLC7A11 protein and mRNA levels were confirmed by Western blot and quantitative RT-PCR (Figures 6A,B,D, *p* < 0.05). We found that fostered *SLC7A11* expression upregulated significantly *NRF2* mRNA (Figure 6E, *p* < 0.05). Furthermore, increased NRF2 protein levels were accompanied by enhanced levels of SLC7A11 (Figures 6A,C, *p* < 0.05). We then analyzed the *VEGFA* mRNA levels of *SLC7A11*^*OE*^ cells. Noteworthy, *SLC7A11*^*OE*^ cells expressed lower amounts of *VEGFA* compared to control cells (Figure 6F, *p* < 0.05). To test the sensitivity to ferroptosis of *SLC7A11*^*OE*^ cells, we treated ARPE19 cells with ferroptosis inducer erastin and GPX4 inhibitors 1S, 3R-RSL3 (RSL3). *SLC7A11*^*OE*^ cells were resistant to erastin and RSL3 compared to control cells (Figures 6G,H and Supplementary Figure 3, *p* < 0.05). To compare the cell viability between *SLC7A11*^*OE*^ and anti-VEGF treatment, ARPE19 treated with anti-VEGF or *SLC7A11*^*OE*^ were perform*ed sim*ultaneously. As shown below, there is no significant difference between anti-VEGF treatment and NC group, which means anti-VEGF treatment has no effects on the ferroptosis (Figure 6I, *p* < 0.05). Altogether, these results demonstrate that SLC7A11 confers a ferroptosis-resistance phenotype in RPE cells and targeting SLC7A11 provides a method for increasing ferroptotic susceptibility in RPE cells.

### SLC7A11 Inhibition Promotes the Development of CNV

**FIGURE 7:**
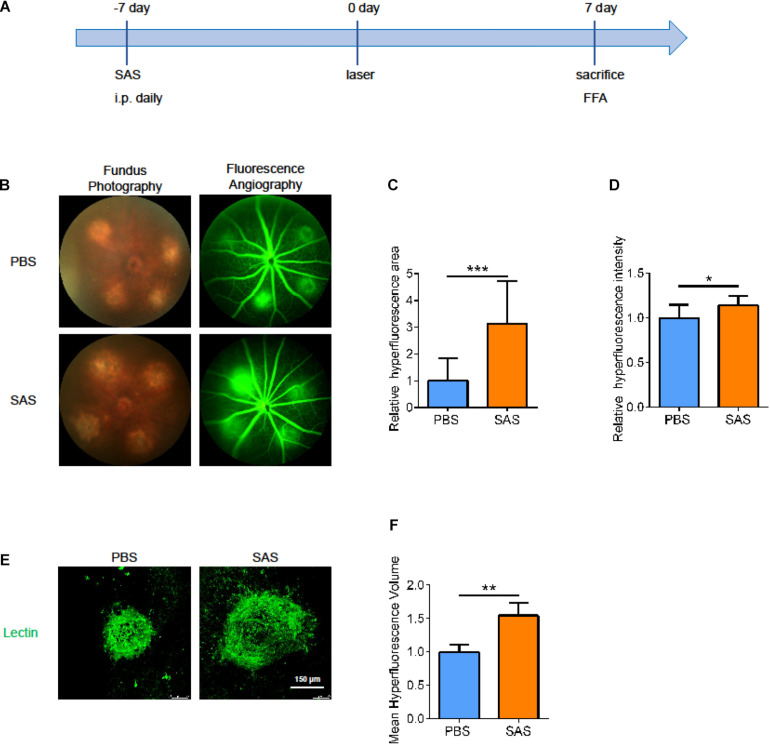
The effect of SLC7A11 inhibitors on CNV areas. **(A)** Schematic diagram of mice administrated SAS. **(B)** Fluorescence imaging of mice after SAS treatment. **(C,D)** The average fluorescence intensity and leakage volume of fluorescence imaging after SAS treatment by Image-J (*n* = 6). **(E,F)** Laser spot size after SAS treatment by immunofluorescence and Image-J (*n* = 6). Bar graphs show mean ± SD. *P*-values: *t*-test (***p < 0.001, **p < 0.01, *p < 0.05). Scale bar = 150 μm.

To further verify the role of SLC7A11 in the process of neovascularization, we intraperitoneally injected SAS or PBS once a day, from 7 days before laser treatment to 7 days after laser treatment. At 7 dpi, we performed fluorescence angiography and isolated choroid for immunofluorescence (Figure 7A). After SAS-treatment, the CNV area was larger than PBS group in both fluorescence angiography and immunofluorescence (Figures 7B–F, *p* < 0.05). This implies that SLC7A11 plays a protective role in CNV progress.

### Regulatory Relationship Between NRF2 and SLC7A11

We found a complex regulatory relationship between SLC7A11 and NRF2, the major antioxidant transcription factor. Considering that after SLC7A11 knocked down or overexpressed, the expression level of NRF2 also changed, so we speculated that the expression of SLC7A11 could regulate the expression of NRF2.

**FIGURE 8:**
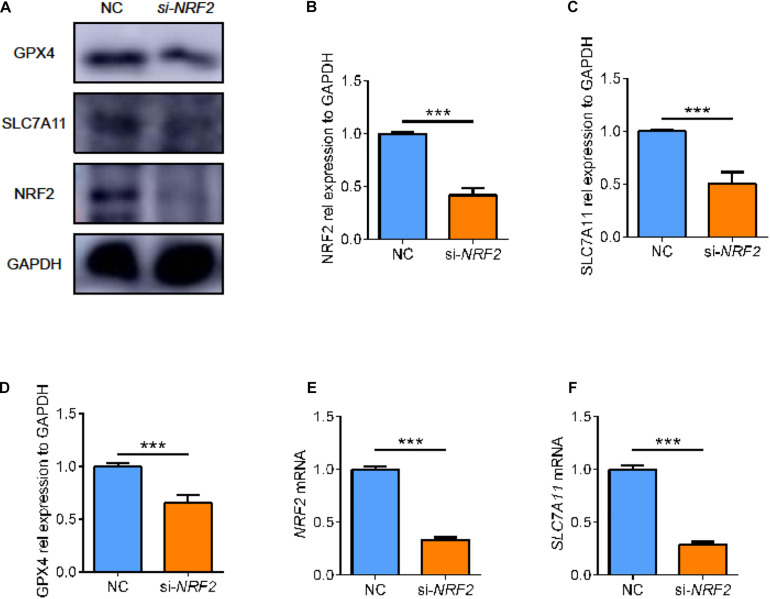
The effect of *NRF2* knockdown on ARPE cell line. **(A–D)** The protein expression level of NRF2, SLC7A11, and GPX4 using siRNA (*n* = 3). **(E,F)** The mRNA expression level of *NRF2* and *SLC7A11* using siRNA (*n* = 6). Bar graphs show mean ± SD. *P*-values: *t*-test (***p < 0.001).

On the other hand, some studies supported NRF2 as one of the upstream regulators of SLC7A11. Therefore, we used small interfering RNA to knock down the expression of *NRF2* in ARPE19 (Figures 8A,B,E, *p* < 0.05). After knocking down *NRF2*, we found that the expression of SLC7A11 decreased, which comported with previous studies (Figures 8A,C,F, *p* < 0.05) (Liu et al., 2017; Shin et al., 2017). Moreover, the lower level of NRF2 was accompanied by a decrease of GPX4 (Figures 8A,D, *p* < 0.05). This suggests that NRF2 and SLC7A11 form a positive feedback loop; they strengthen each other, regulating the downstream GPX4 and ferroptosis.

## Discussion

In clinical practice, AMD imposes a huge economic burden on society because of its damage to eyesight. Treatment with anti-vascular endothelial growth factor (anti-VEGF) has greatly improved the vision of AMD patients and their quality of life. However, there are individual differences in the treatment of anti-VEGF (Amoaku et al., 2015; Yang et al., 2016). Despite the more active targeted therapy, some AMD patients still show an unremitting decline in visual acuity accompanied by the aggravation of macular morphology. In addition, many AMD patients require long-term anti-VEGF treatment, and repeated intraocular injections may cause atrophy of the retina and choroid, which also causes vision loss (Gemenetzi et al., 2017). We hoped to find a general cellular mechanism to explain neovascularization in the course of AMD to find a solution for non-responding patients and patients who need repeated injections to address the deficiencies of anti-VEGF.

Previous studies have shown that in neovascular AMD, RPE and other cells produce VEGF in a hypoxic environment, which promotes the formation and expansion of neovascularization (Bhutto and Lutty, 2012). RPE absorbs excess light and devours the outer section of the photoreceptor filled with unsaturated fatty acids. Thus, RPE consumes a large amount of oxygen and it requires RPE to reserve sufficient capacity to resist oxidative stress. With the increase of age, RPE accumulates too much active oxygen, and its ability to resist oxidative damage decreases. Previous studies have shown that RPE undergoes necrosis and apoptosis under oxidative stress (Hanus et al., 2015). Recent research has shown that RPE cells induced ferroptosis because of the effects of glutamic acid (Sun et al., 2018) and oxidative stress (Totsuka et al., 2019; Lee et al., 2020). However, the ferroptosis of RPE cells *in vivo* is still poorly understood. Our results suggest that SLC7A11 and its-related ferroptosis are involved in the progress of laser-induced CNV. SLC7A11 protects RPE and reduces the CNV area by preventing ferroptosis in RPE.

First, in the laser-induced CNV model, the expression of SLC7A11 in CNV increased, reaching a peak at 7 days. Besides SLC7A11, many molecules related to oxidative stress, including HO-1, GCLC, and NQO1, also peaked at 3 days after the laser treatment. This indicates that the oxidative damage of CNV also peaked at that time. Moreover, VEGFA, closely related to the occurrence and development of CNV, also peaked. It is no doubt that oxidative stress is involved in the formation and expansion of CNV, and our study once again provides conclusive evidence for this. In our *in vitro* experiments, we found that RPE cells expressed more VEGF with increasing H_2_O_2_ concentration. To resist oxidative damage, RPE highly expressed some protective molecules, such as SLC7A11 for compensation.

Second, we also clarified the important regulatory role of SLC7A11 in this process. SLC7A11, as a key component of cystine/glutamate transporter on cell membranes, is an important molecule to buffer oxidative stress injury by regulating the quantity and proportion of intracellular and extracellular GSH (Koppula et al., 2018). In our study, the expression of SLC7A11 increased after laser treatment, and the laser area expanded after using the inhibitor SAS. This illustrates the protective role of SLC7A11 in the progress of CNV. Similarly, ARPE19 expressed more SLC7A11 after being stimulated with H_2_O_2_. As SAS concentration increased, ARPE19 induced ferroptosis by reducing intracellular GSH levels. It also exhibited lower cell viability and higher lipid peroxidation levels.

Third, we explored the mechanism of SLC7A11 protection on RPE. On the 3rd day after laser irradiation, the expression of SLC7A11 increased and there was an increase of GPX4. The high expression of SLC7A11 shows that, after laser treatment, the tissue needs more cystine to enter the cell and synthesize more GSH. GPX4, as an indispensable regulator of ferroptosis, catalyzes the conversion of GSH to GSSG to exert its antioxidant effect (Yang et al., 2014). Therefore, the simultaneous increase of SLC7A11 and GPX4 suggests that the cells were suffering from powerful oxidative stress and required a strong antioxidant capacity. At 7 dpi, the levels of SLC7A11 increased and GPX4 decreased to some extent, and the expression of oxidative stress-related protein also decreased. Therefore, SLC7A11 and GPX4 were consistent with the level of oxidative stress in the environment, indicating that SLC7A11 is sensitive and timely. In ARPE19, we used SAS to suppress SLC7A11 or shRNA to knock it down, and GPX4 showed a consistent downward trend. At the same time, ARPE19 also showed higher lipid peroxidation levels and lower cell viability, implying RPE was undergoing ferroptosis. These results show that SLC7A11 balances the production of effector molecules, and it protects cells from ferroptosis by regulating the expression of GPX4. In oncology, many studies have shown that the inhibition of SLC7A11 is directly related to ferroptosis (Jiang et al., 2015; Lang et al., 2019). But in the eyes, especially neovascular AMD, little is known about the ferroptosis of RPE. Our research fills the gap in the study of ferroptosis in this field, and it proposes some possible mechanisms.

In addition, we found complex interactions between NRF2 and SLC7A11, such that they form a positive feedback loop and strengthen each other to regulate downstream GPX4 and ferroptosis. We used small interfering RNA to knock down NRF2 and found that SLC7A11 fell with it. Interestingly, when we used short-hairpin RNA to knock down SLC7A11, we noticed that NRF2 showed a corresponding decline. Conversely, SLC7A11 overexpression induced higher expression of NRF2 compared to control cells. Previous studies have noted that NRF2 regulates the expression of SLC7A11, but we found a change that SLC7A11 can also affect the expression of NRF2. This has been confirmed by our study. We speculated that the positive feedback regulation between NRF2 and SLC7A11 may function as protection against oxidative stress. More specific regulatory mechanisms for NRF2 and SLC7A11 require more attention.

However, there were some aspects of the experiment worth improving. We lacked more detailed verification and explanation to further prove the complex relationship between NRF2 and SLC7A11.

In conclusion, our study proposed a possible new mechanism in RPE in the laser-induced CNV model, namely SLC7A11 and its suppressed ferroptosis. SLC7A11 could play an antioxidant role, protect cells from ferroptosis, and reduce CNV areas by activating or increasing GPX4. It provides a new therapeutic idea for neovascular AMD patients who are clinically insensitive to anti-VEGF treatment or who require repeated injections that cause side effects. Delivery of target gene DNA to specific tissue by AAV is powerful technology in the field of gene therapy. And in the future, delivery of SLC7A11 to RPE by AAV would become a possible clinical method to protect the cell from ferroptosis in the neovascular AMD patients.

## Data Availability Statement

The original contributions presented in the study are included in the article/Supplementary Material, further inquiries can be directed to the corresponding authors.

## Ethics Statement

The animal study was reviewed and approved by the Association for Research in Vision and Ophthalmology Statement for the Use of Animals in Ophthalmic and Vision Research. Written informed consent was obtained from the owners for the participation of their animals in this study.

## Author Contributions

XZ, MG, and JL conceived the study, designed the experiments, and performed the experiments with the help of YC, YiW, YuW, YX, and ZZ. XZ wrote the manuscript. XW, MJ, XL, FW, and XS interpreted the data and contributed to discussion. XS and FW were the guarantor of this work and as such, had full access to all the data in the study and took responsibility for the integrity of the data and the accuracy of the data analysis. All authors reviewed and concurred with the final manuscript.

## Conflict of Interest

The authors declare that the research was conducted in the absence of any commercial or financial relationships that could be construed as a potential conflict of interest.

## Supplementary Material

Supplementary Figure 1 The level of lipid peroxidation and cell death of *SLC7A11*^*KD*^ ARPE19. **(A)** The level of lipid peroxidation of *SLC7A11*^*KD*^ ARPE19 by BODIPY^TM^ 581/591. **(B)** Immunofluorescence for PI staining of *SLC7A11*^*KD*^ ARPE19 with the treatment of Fer-1 (1000 nM) and Lip-1 (20 μM). Scale bar = 50 μm.

Supplementary Figure 2 The level of lipid peroxidation and cell death of ARPE19 after SAS treatment. **(A)** Immunofluorescence for PI staining of ARPE19 with the treatment of Fer-1 (1000 nM) and Lip-1 (20 μM) after SAS treatment. **(B)** The level of lipid peroxidation under SAS stimulation by BODIPY^TM^ 581/591. Scale bar = 50 μm.

Supplementary Figure 3 The cell death of *SLC7A11*^*OE*^ ARPE19 with PI staining. Immunofluorescence for PI staining of *SLC7A11*^*OE*^ ARPE19 with the treatment of erastin (200 μM) or RSL3 (20 μM). Scale bar = 50 μm.
