# Supplementary figures and images for: SLC7A11 Reduces Laser-Induced Choroidal Neovascularization by Inhibiting RPE Ferroptosis and VEGF Production
Source: Front Cell Dev Biol. 2021 Feb 18;9:639851. doi: 10.3389/fcell.2021.639851 (PMC7930391; doi:10.3389/fcell.2021.639851)

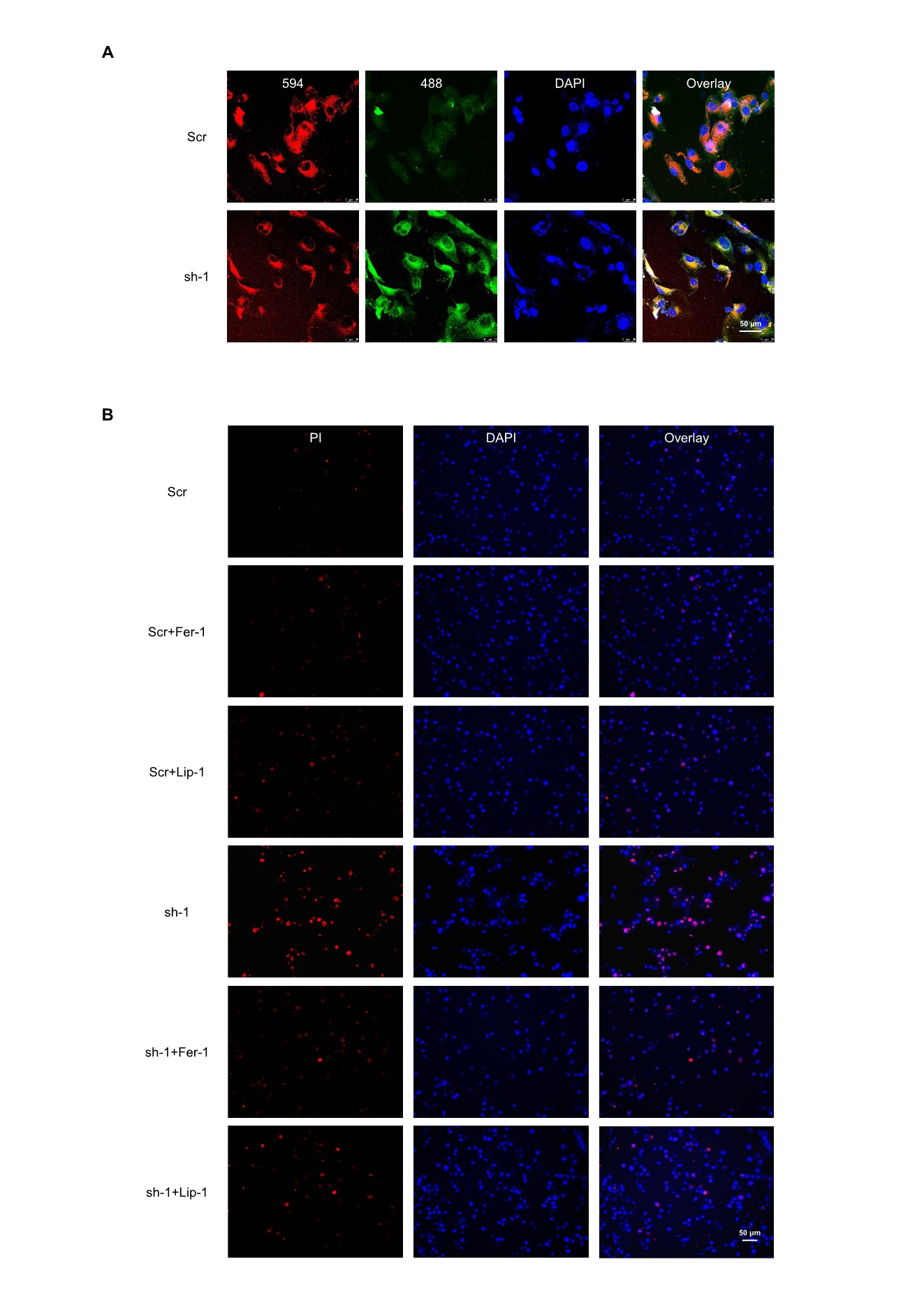

Supplement: Supplementary Figure 1 — The level of lipid peroxidation and cell death of SLC7A11KD ARPE19. (A) The level of lipid peroxidation of SLC7A11KD ARPE19 by BODIPYTM 581/591. (B) Immunofluorescence for PI staining of SLC7A11KD ARPE19 with the treatment of Fer-1 (1000 nM) and Lip-1 (20 μM). Scale bar = 50 μm. [file Image_1.JPEG]

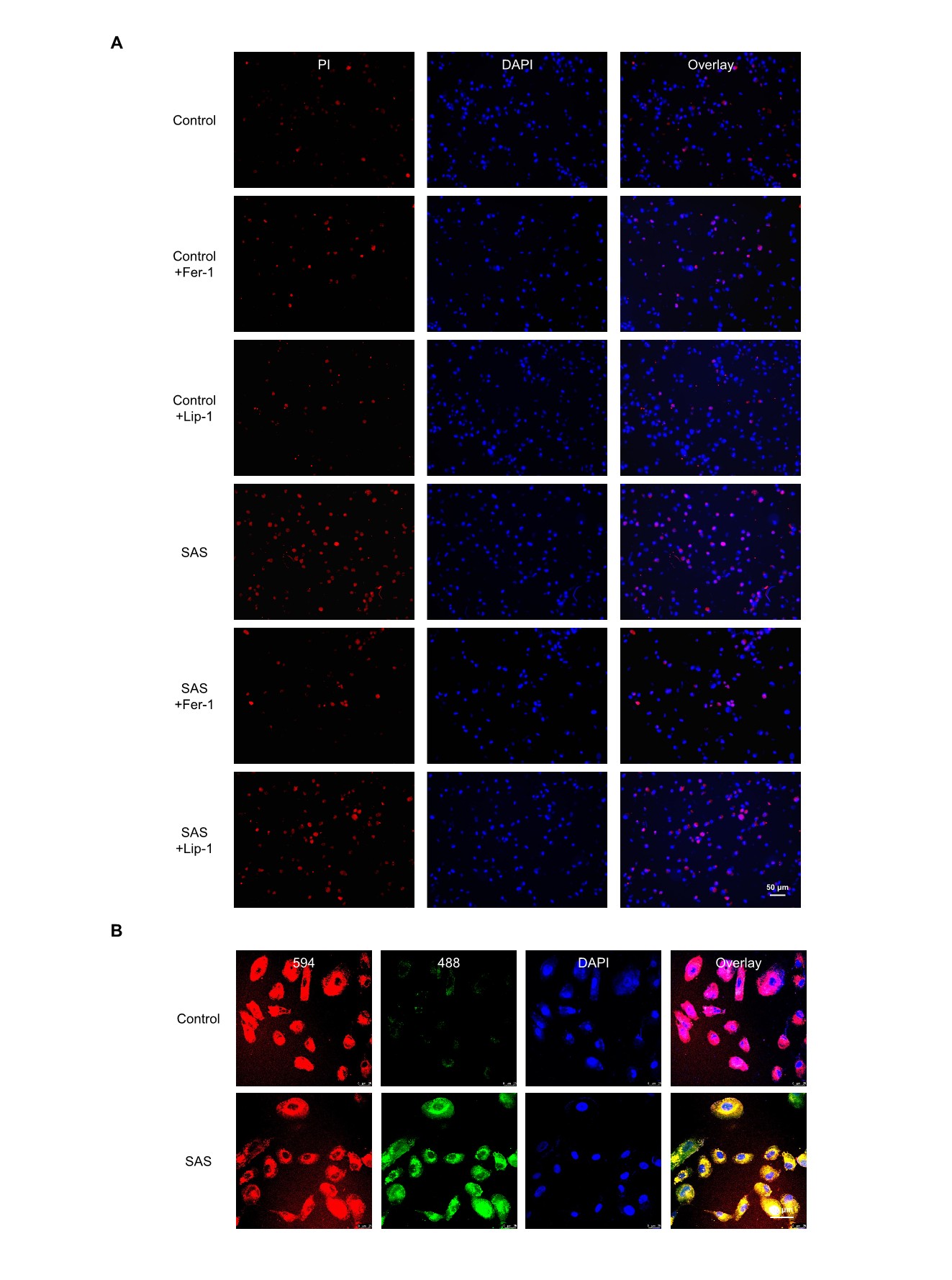

Supplement: Supplementary Figure 2 — The level of lipid peroxidation and cell death of ARPE19 after SAS treatment. (A) Immunofluorescence for PI staining of ARPE19 with the treatment of Fer-1 (1000 nM) and Lip-1 (20 μM) after SAS treatment. (B) The level of lipid peroxidation under SAS stimulation by BODIPYTM 581/591. Scale bar = 50 μm. [file Image_2.JPEG]

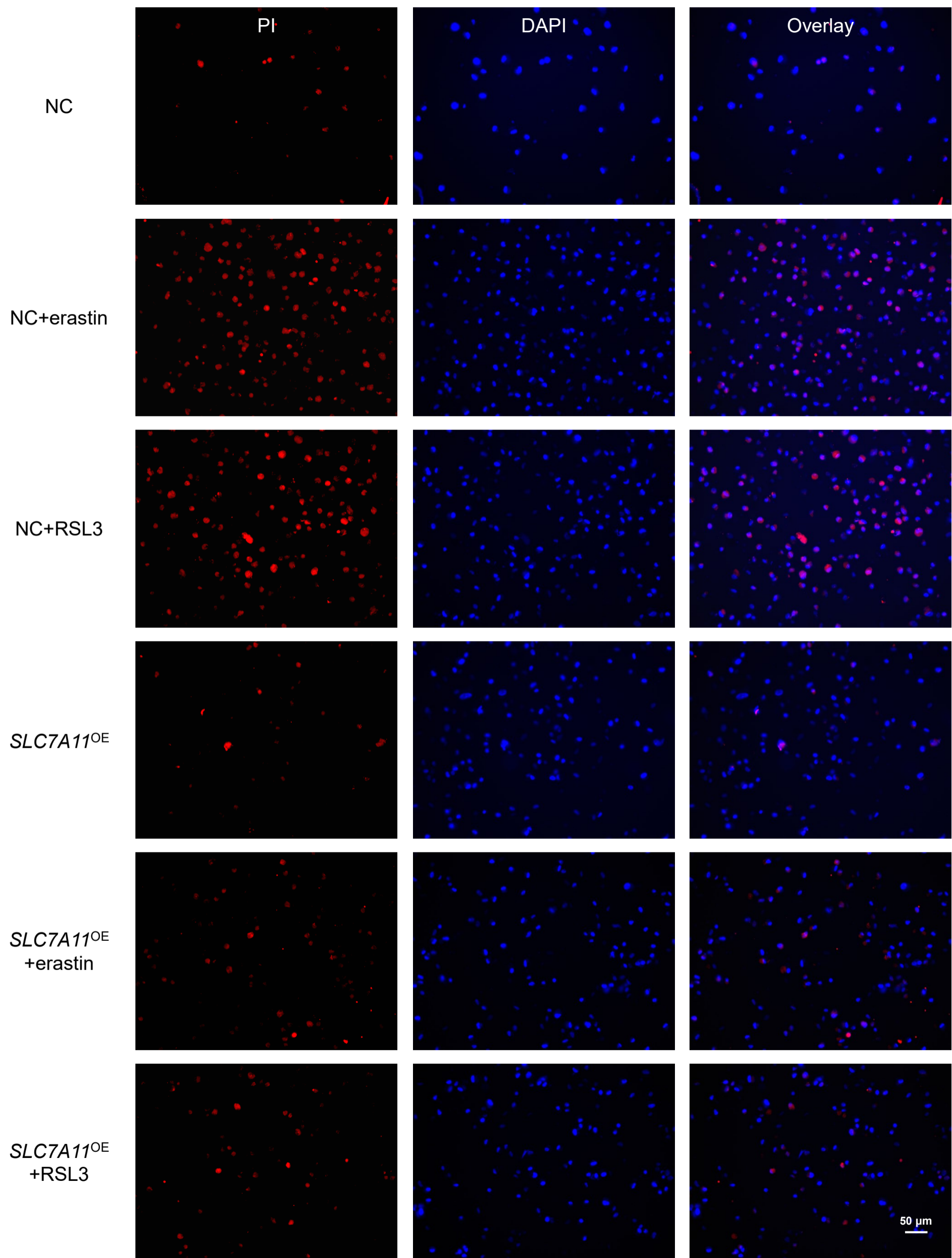

Supplement: Supplementary Figure 3 — The cell death of SLC7A11OE ARPE19 with PI staining. Immunofluorescence for PI staining of SLC7A11OE ARPE19 with the treatment of erastin (200 μM) or RSL3 (20 μM). Scale bar = 50 μm. [file Image_3.pdf]
